# Exercise Training in Heart Failure: Current Evidence and Future Directions

**DOI:** 10.3390/jcm14020359

**Published:** 2025-01-09

**Authors:** Loay Eleyan, Ahmed R. Gonnah, Imran Farhad, Aser Labib, Alisha Varia, Alaa Eleyan, Abdullah Almehandi, Abdulrahman O. Al-Naseem, David H. Roberts

**Affiliations:** 1 Leeds Teaching Hospitals NHS Trust, Leeds LS1 3EX, UK; loayeleyan@gmail.com; 2 Imperial College Healthcare NHS Trust, London W2 1NY, UK; ahmedgonnah18@outlook.com; 3 School of Medicine, University of Liverpool, Liverpool L69 3GE, UK; hlifarha@liverpool.ac.uk (I.F.); hlavaria@liverpool.ac.uk (A.V.); 4 Sheffield Teaching Hospitals NHS Trust, Sheffield S10 2JF, UK; aser.labib@nhs.net; 5 School of Medicine, University of Manchester, Manchester M13 9PL, UK; alaa.eleyan@student.manchester.ac.uk; 6 Institute of Cardiovascular Sciences, University College London, London WC1E 6DD, UK; abdullah.almehandi.24@ucl.ac.uk; 7 Department of Surgery, Jaber Al-Ahmad Hospital, Kuwait City 45706, Kuwait; abdulrahman.alnaseem.22@ucl.ac.uk; 8 Lancashire Cardiac Centre, Blackpool FY3 8NP, UK

**Keywords:** exercise training, HFpEF, HFrEF, pVO_2_, 6-min walk test

## Abstract

Heart Failure (HF) is a prevalent condition which places a substantial burden on healthcare systems worldwide. Medical management implemented with exercise training (ET) plays a role in prognostic and functional capacity improvement. The aim of this review is to determine the effect of exercise training (ET) on HFpEF and HFrEF patients as well as exercise modality recommendations in frail and sarcopenic subpopulations. Pharmacological therapy structures the cornerstone of management in HF reduced ejection fraction (HFrEF) and aids improved survival rates. Mortality reduction with pharmacological treatments in HF preserved ejection fraction (HFpEF) are yet to be established. Cardiac rehabilitation (CR) and ET can play an important role in both HFrEF and HFpEF. Preliminary findings suggest that CR significantly improves functional capacity, exercise duration, and quality of life. ET has shown beneficial effects on peak oxygen consumption (pVO_2_) and 6 min walk test distance in HFrEF and HFpEF patients, as well as a reduction in hospitalisation and mortality rates; however, the limited scope of larger trials reporting on this underscores the need for further research. ET also has been shown to have beneficial effects on depression and anxiety levels. High-intensity training (HIT) and moderate continuous training (MCT) have both shown benefits, while resistance exercise training and ventilatory assistance may also be beneficial. ET adherence rates are higher when enrolled to a supervised programme, but prescription rates remain low worldwide. Larger robust trials are required to determine ET’s effects on HF, as well as the most efficacious and personalised exercise prescriptions in HF subtypes.

## 1. Introduction

Heart failure (HF) is a multifaceted and prevalent condition affecting over 64 million individuals worldwide and standing as a leading cause of hospitalisation and mortality, thus posing a significant public health challenge [1]. Its prevalence ranges from 1 to 2%, with a notable increase in the older population, surpassing 10% among those aged 70 and above [2,3]. HF ESC classification is usually based on ejection fraction (EF): preserved, >50% (HFpEF); mid-range, 41–49% (HFmrEF); reduced, <40% (HFrEF); and improved, 10-point increase from baseline with a subsequent ejection fraction exceeding 40% (HFImpEF) [4,5,6]. Women generally have a higher incidence of HFpEF. Developed countries have witnessed a decline in the incidence of HFpEF and HFrEF, primarily attributed to advancements in HF management, with HFrEF showing a more significant decrease (−45%) compared to HFpEF (−28%) [7]. The management of HFpEF patients mainly focuses on symptomatic care as opposed to HFrEF, highlighting the need for additional therapeutic modalities to treat and manage this sub-group of HF patients. Exercise training (ET) is perceived to be a known component of HF management [6]. The existing literature focuses on either HFpEF or HFrEF patients separately without comparing the effectiveness of ET across both subtypes. This review highlights the emerging role of ET for treating chronic heart failure patients with HFpEF and HFrEF. The exploration of both groups allows for a comparative analysis of how ET impacts the clinical outcomes of patients within different sub-types of HF. Furthermore, efficacious exercise modalities have been under-reported in frail and sarcopenic subpopulations. In this paper, we review the effect of ET on HFpEF and HFrEF patients, as well as frailty and sarcopenic subtypes, and provide guidance on ET implementation.

## 2. Pathophysiology

**Figure 1:**
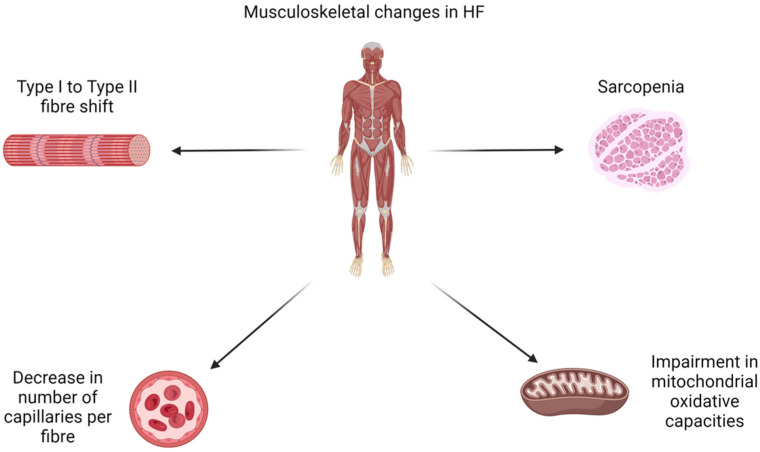
Musculoskeletal changes in HF.

Sarcopenia is found in 20–50% of HFrEF patients and contributes directly to frailty, exercise intolerance, and early fatigue. It poses a significant impact on daily activities and is associated with increased morbidity and mortality [8,9,10]. HFrEF patients have more pronounced molecular alterations within skeletal myocytes, including a shift in muscle fibre type from slow to fast twitch fibres (type I to type IIb) [11]. This could be due to the reduction in PGC-1α (peroxisome proliferator-activated receptor gamma coactivatory-1 alpha), a key regulator for type 1 fibre formation [12]. A decrease in the number of capillaries per fibre is also seen in both HFrEF and HFpEF patients, implying a less efficient system of diffusion from blood to myocyte [11]. HFrEF and HFpEF have both been linked with rapid depletion of high-energy phosphate, as well as impairment in mitochondrial oxidative capacities [11]. The contribution of all these factors, as well as the presence of a systemic inflammatory response, leads to muscle atrophy, exercise intolerance, and a decrease in quality of life [11]. An overview of the musculoskeletal changes in HF is visualised in Figure 1.

## 3. Functional Assessments of Heart Failure

The New York Heart Association (NYHA) classification helps gauge the clinical status of HF patients, but functional capacity assessments help to determine exercise tolerance. Measuring peak oxygen consumption (VO_2_) using cardiopulmonary exercise testing (CPET) is generally considered to be the best indicator for endurance and fitness and is associated with indices of health-related quality of life (HRQoL) [13]. Abnormal CPET have been shown in both HFpEF and HFrEF patients, with HFrEF patients exhibiting relatively low peak oxygen uptake (pVO_2_) and elevated VE/VCO_2_ slopes, a sign of poor ventilatory efficiency due to more ventilation requirement to remove a given amount of CO_2_ [14].

The 6 min walking test distance (6MWTD) is a simpler and reproducible alternative to CPET and has proven useful in assessing functional capacity and clinical outcomes in HF patients [6,14,15]. Several trials have also reported that 6MWTD can evaluate the efficacy of different treatments [16]. The distance walked is associated with improved survival rates in univariate analyses, but not in multivariate analyses, which highlights the importance of the consideration of other factors such as peak VO_2_ and NYHA class [17]. Mortality has been shown to be higher in those who manage to walk < 350 m [18].

HF patients are at increased risk of frailty in comparison to the general population [19,20], which equates to higher hospitalisation rates, longer hospital stays, and increased mortality [21,22,23]. Frailty indexes can be used to quantify risks among HF patients using variables such as symptoms, disabilities, and laboratory abnormalities [24]. BNP and NT-proBNP play an important role in the diagnosis and monitoring of HF patients [25]. Reductions in these biomarkers following aerobic and/or resistance exercise training are associated with functional improvements in patients with HFrEF, and they have been used to objectively monitor patients’ responses to cardiac rehabilitation and ET programmes [25].

## 4. Optimising Functional Capacity in Patients with Heart Failure

Angiotensin-converting enzyme inhibitors (ACE-I), angiotensin receptor-neprilysin inhibitors (ARNI), beta blockers, mineralocorticoid receptor antagonists (MRA), and sodium–glucose co-transporter 2 (SGLT2) inhibitors all improve survival, alleviate symptoms, reduce hospitalisations, and improve physical function in patients with HFrEF [26,27,28,29,30]. Despite optimal guideline-directed medical therapy (GDMT), there has been a notable increase in the incidence and prevalence of HF-related hospitalisations [1,2,3].

Histological examination of skeletal muscle in patients with HF show reduced oxidative capacity, reduced volume density, and reduced surface area of mitochondrial cristae caused by insufficient blood flow during exercise [31]. Trials in patients with HFrEF have shown that after treatment with an ACE-I, the inability of peripheral vessels to dilate was reversed, increasing blood flow and, in turn, improving oxidative capacity and ultimately exercise tolerance [32]. After 6 months of ARNI treatment in patients with HFrEF, cardiopulmonary test variables improved; peak VO_2_ increased from 15.8  ±  3.4 to 17.0  ±  4.0 mL/kg/min and O_2_ pulse increased from 11.5  ±  2.5 to 12.6  ±  2.4 mL/beat [33]. However, an observational study on Sacubitril/valsartan use in 13 HFrEF patients demonstrated no significant peak VO_2_ increase (*p* = 0.49) in comparison to patients on optimal standard medical therapy [34]. Thus, larger trials are required to demonstrate the effects of ARNI on CPET parameters. ARNI treatment also demonstrated reduced NT-proBNP levels, increased ejection fraction, and improved NYHA class [35]. Similarly, MRA treatment improved exercise capacity in patients with HFrEF [36]. In HFpEF, pharmacological treatments primarily focus on symptom management (e.g., diuretics for fluid overload) and addressing underlying causes [6,37,38,39]. MRA appears to have no effect on peak VO_2_, 6MWD, or QoL in HFpEF [24]. Conversely, beta blocker withdrawal in HFpEF exhibited a significant increase in peak VO_2_ band or peak VO_2_% compared to controls [40], associated with a significant improvement in QoL [40].

Iron-deficiency anaemia is recognised in patients with HFrEF and is associated with reduced exercise capacity [41]. Improvement is seen in pVO_2_, exercise capacity, and QoL following iron infusions [41,42]. Evidence in HFpEF remains uncertain, although improved exercise capacity has been noted, explained by a reduced production in oxygen radical species, leading to improvement in diastolic function as well as enhanced endothelial function [41].

There are limitations to who can receive the therapy due to the availability of treatment, and hence clear guidelines are warranted to commission this treatment option to patients with worsening heart failure control despite optimal medical therapy.

## 5. The Role of Cardiac Rehabilitation Programmes

Cardiac rehabilitation programmes play a significant role in patients with HF. Guidelines from the American College of Cardiology (ACC), the American Heart Association (AHA), and the European Society of Cardiology (ESC) recommend CR for HF patients to enhance physical activity and QOL, especially for those with severe disease and comorbidities [6,37,42,43,44,45,46]. A multidisciplinary specialist team is involved in the assessment and referral for rehabilitation. A typical cardiac rehabilitation programme includes medical evaluation, education on medication adherence, dietary advice, psychosocial support, and physical activity counselling [47,48]. CR should be provided in a convenient setting (at home, in the community, or in the hospital).

NICE guidelines recommend exercise training (ET) as an adjunct to medical therapy in patients with HF based on the results of trials such as HF-ACTION [43,44,47]. HFrEF patients who have already established on optimal GDMT should also be considered for enrolment. In patients with HFpEF where the efficacy of GDMT remains uncertain, ET can also be considered [47]. Structured exercise training includes high-intensity training (HIIT) or moderate continuous training (MCT) carried out for varying lengths of time. A personalised exercise training programme should be considered during hospital admission and include an assessment appointment within 10 days of discharge from hospital. It should include clear and concise education and information surrounding exercise, including its effects on psychological wellbeing [47].

## 6. Efficacy of Exercise Training in Patients with HFpEF

Mueller et al. found that exercise training in patients with HFpEF improved peak oxygen uptake (pVO_2_) by 8.0% ± 15.7%, whereas the control group experienced a reduction of 2.0% ± 18.3% (*p* = 0.001) [49]. There was a strong correlation between increased exercise training and elevated pVO_2_ at 4 months and at 1 year follow-up [45,50]. Supervised high-intensity interval training (HIIT) and moderate continuous training (MCT) have been analysed for their impact on pVO_2_ [13,46]. HIIT aimed to use 80–90% of heart rate reserve (the difference between a person’s maximum heart rate and their resting heart rate) during four-minute intervals separated by three minutes of recovery, while MCT only used 35–50% of the heart rate reserve five times a week during 40 min sessions [49]. MCT was found to be the most effective training modality, displaying an increase in pVO_2_ of 1.6 (2.5) mL/kg/min at 3 months compared to 1.1 (3.0) mL/kg/min for HIIT and −0.6 (3.3) mL/kg/min for the control group (*p* = 0.002) [49]. On the contrary, another randomised control trial highlighted the superiority of HIIT, which showed an increase in pVO_2_ by 3.5 (3.1–4.0) compared to 1.9 (1.2–2.5) for MCT at 3 months (*p* < 0.001) [46]. This discrepancy underscores the necessity for determining the best training modality for individual patients. Despite this inconsistency, diastolic function achieved significant improvement regardless of which training method was used [46].

The effect of ET has also been evaluated using the 6MWTD. A multicomponent behavioural exercise intervention focussed on improving clinical outcomes in HFpEF demonstrated significant increases in walking distance compared to the enhanced usual care group (EUCG) at 6, 12, and 18 months [51]. The EUCG patients were provided with paid access to a fitness centre to exercise independently without the behavioural support and exercise coaching provided. HFpEF patients undergoing ET also showed enhanced walking distances compared to the control group (*p* < 0.001) [51] when required to perform at least 120 min of moderate-intensity exercise per week (using a heart rate monitor to ensure exercise intensity stayed within 40–80% of heart rate reserve) with significant results at 12 and 18 months of follow-up [51]. Pandey et al. compared MCT in both HFpEF and HFrEF patients, revealing a significant increase in peak oxygen consumption in the HFpEF group (18.7 ± 17.6%) compared to the HFrEF group (−0.3 ± 15.4%) (*p* < 0.001), although changes in 6MWTD were not statistically significant [52,53].

Data on the impact of ET on hospitalisation rates and mortality in HFpEF patients are limited. However, a pilot study of 50 patients reported fewer hospitalisations in the ET group [54]. Additionally, a cardiac rehabilitation programme involving 85 patients showed lower hospitalisation and lower cardiovascular mortality in the exercise intervention group (11%) compared to the control group (24%) at 6 months follow-up. At 18 months, there was no significant difference between the intervention group (25%) and the control group (29%); however [55], these studies’ small sample sizes indicate the need for further large-scale research to determine the effects of ET. Table 1 summarises the trials on the efficacy of exercise training in HFpEF patients.

## 7. Efficacy of Exercise Training in Patients with HFrEF

The efficacy of exercise training has been evaluated more widely in HFrEF patients. Similar to HFpEF patients, ET has been shown to improve peak oxygen consumption in patients with HFrEF. In the HF-ACTION trial, which included 2331 HFrEF patients assigned to either an ET group or a usual care group (UCG), the ET group exhibited significantly better improvements in pVO_2_ at both 3 months and 12 months compared to the UCG [43]. The ET group had a significant improvement in 6MWTD at 3 months, but this difference was not significant at 12 months [43]. The decline in 6 min walk test distance at 12 months may result from decreased adherence to exercise, disease progression, worsening comorbid conditions, medication side effects, or lifestyle changes. The need for ongoing adjustments to the exercise programme may also play a role in the reduced benefits observed over time.

Different exercise modalities have also been examined for their impact on pVO_2_. A 3 week interval training programme demonstrated a 21% increase in pVO_2_ in the high-intensity interval training (HIIT) group (n = 16) compared to a 5% increase in the moderate continuous training (MCT) group (n = 15) (*p* = 0.009) [56]. Additionally, a multi-centre trial with 215 patients randomly assigned to HIIT, MCT, or regular exercise (RRE) for 12 weeks found significant improvements in pVO_2_ for both HIIT (1.4; 0.2–1.6, *p* = 0.02) and MCT (1.8; 0.5 to 3.0, *p* = 0.003) compared to the RRE group, although no significant differences were noted between HIIT and MCT (−0.4; −1.7 to 0.8, *p* = 0.70) [57].

The Aristos-HF trial, which included 88 HFrEF patients, demonstrated improved pVO_2_ across various exercise modalities, including aerobic training (AT- MCT and HIIT), AT and resistance training (RT), AT and inspiratory muscle training (IMT), and ARIS (a combination of AT + RT + IMT) for 180 min/week for 12 weeks, with the greatest increase seen in the ARIS group [58]. The Aristos-HF trial also found the greatest improvement in 6MWTD in the ARIS group (55.2 [27.6 to 82.7], *p* ≤ 0.001) [58]. A 4-month non-randomised study of combined resistance and endurance training involving 27 HFrEF patients showed a significant increase in pVO_2_ compared to an untrained group (n = 22) [59].

ET has also been shown to improve left ventricular ejection fraction and reduce left ventricular end-diastolic dimension (LVEDD) in HFrEF patients. In a 3-week interval training programme, there was an increase in LVEF in the HIIT group (3.3%, *p* = 0.034), although intergroup changes between HIIT and MCT were non-significant compared to each other [56]. Another trial evaluating LVEDD displayed a significant difference in LVEDD reduction in the HIIT group compared to the regular exercise group (−2.8 mm; −5.2 to −0.4 mm; *p* = 0.02), but not between HIIT and MCT (−1.2 mm; −3.6 to 1.2 mm; *p* = 0.45) [57]. NT-proBNP levels, another parameter analysed in HFrEF patients, showed no significant difference between the HIIT, MCT, and RRE regimens [56]. The Aristos-HF trial found the greatest improvement in left ventricular end-systolic diameter (LVESD) in the ARIS group, with all interventions improving left ventricular end-diastolic dimension (LVEDD) and LVEF [58].

Mortality and hospitalisation rates following ET in HFrEF patients have been insufficiently reported. The HF-ACTION trial found no significant difference in mortality rates between the ET group (16%) and UCG (17%) at a median follow-up of 30 months (*p* = 0.70), and all-cause hospitalisation rates were also similar (*p* = 0.79) [43]. Conversely, a home-based cardiac rehabilitation (CR) randomised control trial reported lower hospitalisation rates in the CR group (5%) compared to the control group (14%), reducing readmission rates by nearly 10% over 12 weeks [60]. Further studies are necessary to determine the effect of ET on hospitalisation and mortality rates.

**Figure 2:**
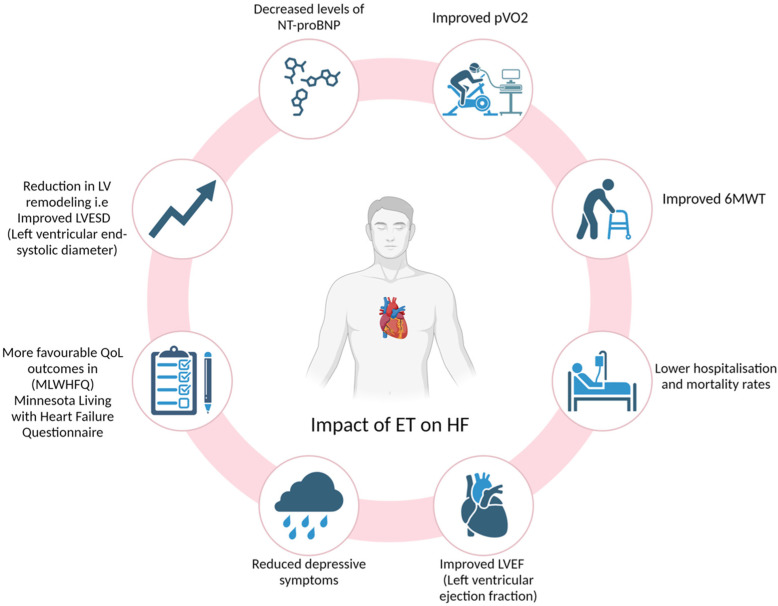
Impact of ET on HF.

**Figure 3:**
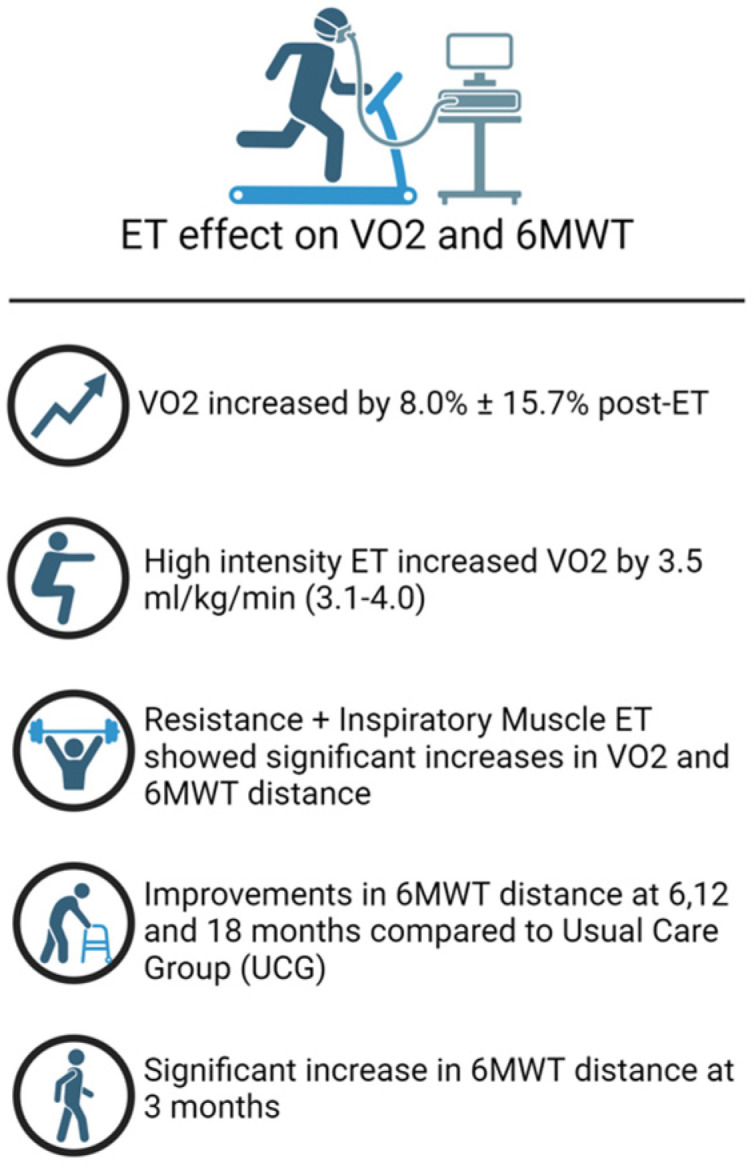
ET effect on VO_2_ and 6MWT.

Patients with elevated levels of NT-proBNP exhibit higher risks of cardiovascular disease and mortality regardless of previous cardiovascular history, and combined training showed a significant reduction in NT-proBNP levels [59,61,62,63,64,65]. Finally, higher levels of physical activity have been associated with a decrease in cardiovascular disease events. In a multivariable model, increased physical activity was linked to a reduced incidence of CVD events (HR: 0.802; 95% CI: 0.719–0.896; *p* < 0.001) [64]. Routine physical activity has further shown a reduction in biomarkers of systemic inflammation, which are present in progressive HF [65]. Figure 2 presents the impact of ET on HF patients, while Figure 3 looks at the effect ET exhibits on VO_2_ and the 6MWT. The summaries of the trials on the efficacy of exercise training in HFrEF patients can be seen in Table 2.

## 8. Resistance Exercise Training Modality

Changes in muscle function and composition are important determinants in the prognosis of HF patients. The effectiveness of resistance exercise training (RET) has been evaluated in patients with sarcopenia. It usually involves muscle contraction against external resistance, with the aim of increasing muscle strength, tone, or mass. One trial focused on a 12 week resistance training programme in ten stable HF patients and demonstrated a marked increase in quadricep strength (*p* < 0.01), but not muscle mass (*p* > 0.2) [53]. A randomised control trial of RET and nutritional supplementation on muscle size and strength in 94 generally frail patients (not specifically HF patients [66]) showed that muscle strength and the size of hip and knee extensors improved significantly (*p* = 0.001), improving mobility and physical activity levels [66]. RET may help frail HF patients adjust and take part in aerobic training; however, further studies are required with larger, more robust trials to highlight the extent of which ET is applicable here.

## 9. Ventilatory Assistance

Ventilatory assistance, the use of mechanical pressure support to aid breathing, has been shown to reduce ventricular preload and afterload and to improve the work of breathing in decompensated HF patients. This involves improving the oxygenation of myocardial tissue and optimising cardiac output requirements [67]. A randomised control trial including stable chronic HF patients studied the effect of ventilatory assistance on exercise endurance [68]. Exercise time improved significantly with pressure support in comparison to the control group (*p* = 0.004). In contrast, CPAP only produced a small improvement in exercise time (*p* = 0.079) [68]. The positive effects of ventilatory assistance could be applied in patients unable to adhere to ET; however, once again, further robust research with large trial groups is necessary to ascertain the viability of this option.

## 10. Exercise Training and Quality of Life in Patients with Heart Failure

Quality of life for HF patients was assessed using the Minnesota Living with Heart Failure Questionnaire (MLWHFQ), the Kansas City Cardiomyopathy Questionnaire (KCCQ), and the Short Form Health Survey (SF-36), among others [37,69]. The MLWHFQ consists of 21 questions scaled 0–5 to indicate the effect of heart failure during the previous 4 weeks. The final score is the summation of all responses. This questionnaire reflects the impact of heart failure on patients’ lives in the physical, emotional, and socioeconomic domains [70]. The KCCQ contains 23 items, covering 6 domains—symptoms, physical functions, QoL, social limitations, self-efficacy, and symptom stability, as well as two summary scores—a clinical summary, and an overall summary for a total scale of 0–100 [71]. As the name suggests, the SF-36 consists of 36 questions with 8 weighted domains—vitality, physical function, bodily pain, general health perception, physical role, emotional role, social role, and mental health—for a total score between 0 and 100 [72]. These questionnaires were used when assessing patients’ responses to exercise ET.

## 11. Studies Assessing QOL Following ET in Patients with HFpEF

A study involving 116 HFpEF patients used the MLWHFQ and the SF-36 questionnaires. Post-intervention, MLWHFQ scores were lower in the ET group (23.13, 95% CI, 18.32–27.95) compared to the control group (CG) (28.20, 95% CI, 23.61–32.79). SF-36 showed better outcomes for the ET group in the domains of role—physical (*p* < 0.05), vitality (*p* < 0.05), and role—emotional compared to the CG. Interestingly, no significant association was found between pVO_2_ or 6MWTD and HRQoL changes in the ET group, however [50]. This may be due to the patient-centred nature of HRQoL assessments, which capture the emotional and psychological benefits not always reflected in physical measurements. Individual responses, assessment timing, and the complex interplay of physical and non-physical factors may contribute to this disconnect. Thus, improvements in QoL can occur independently of changes in physical fitness.

Two studies used the KCCQ [49,51]. One found no significant change in QoL after 3 months between high-intensity interval training (HIT), moderate continuous training (MCT), and CG. However, after 12 months, the MCT group reported significantly higher QoL outcomes compared to the CG (11, 95% CI, 2–19). No significant difference was observed between the HIT group and the CG after 12 months (4, 95% CI, −3 to 12) [49]. Another study indicated that KCCQ scores improved significantly in the home-based care ET group (HCG) after 18 months compared to the usual care group [51].

Further studies assessed the effect of ET on depression in HFpEF. An analysis of the HEART camp group trial demonstrated a statistical improvement in depression post-intervention (*p* = 0.029) [73]. Another study utilising the Hospital Anxiety and Depression Score (HADS) displayed a mild decrease in depression at 6 months (8, 7–9) in comparison to baseline (7.5, 6–8) [55].

## 12. Studies Assessing QOL Following ET in Patients with HFrEF

The Aristos-HF trial demonstrated significant benefits in MLWHFQ scores for the aerobic resistance interval training (ARIS) and aerobic training/inspiratory muscle training (AT/IMT) groups (*p*  ≤  0.0001) [59]. Additionally, a randomised controlled trial involving 33 HFrEF patients who underwent high-intensity interval training (HIIT) and 39 patients who did not participate in exercise training found significant improvements in health-related QoL outcomes in the intervention group. MLWHFQ scores significantly decreased (indicating improved QoL) in the intervention group after 12 weeks, while no change was observed in the control group (intergroup changes, *p* < 0.001) [74].

**Figure 4:**
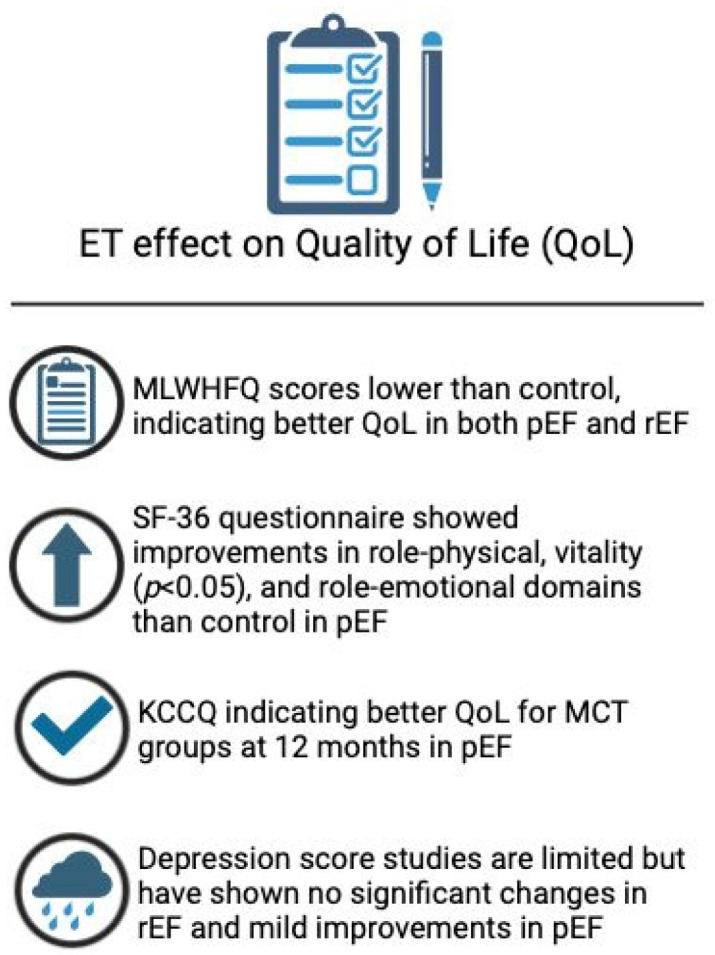
ET effect on QoL.

A multicentre trial reported no significant differences in QoL outcomes between ET and control groups using the KCCQ [58]. The trial also demonstrated no difference in hospital and anxiety scale in HFrEF post-intervention [58], while a study utilising the Zung Depression Rating Scale (ZDRS) found a significant improvement in the ET intervention group (*p* = 0.005), while it remained at a similar score in the control group (*p* = 0.19) [74]. Studies that analysed the effects of ET on depression do not appear to show significant improvement in HFrEF patients. An overview of ET’s effect on QoL can be seen in Figure 4.

The benefits observed in specific QoL measures suggest that personalised treatment approaches and longer-term follow-up are essential, with more RCTs required to confirm the present findings.

## 13. Adherence to Exercise Training

Maintaining adherence to ET can be challenging, and is generally higher in supervised programmes and when personalised strategies are implemented. Current NICE guidelines state that a personalised exercise-based cardiac rehabilitation programme should be offered unless the patients’ condition is unstable [47]. This should include a psychological and educational component and be provided in a format and setting that is easily accessible for the patient [47]. While specific training exercises are not mentioned, consistent adherence and attendance are required to see clinical benefits [75].

## 14. HFpEF Adherence Rates

A study comparing high-intensity interval training (HIT) and moderate continuous training (MCT) in HFpEF patients found high adherence rates at 3 months. Specifically, 80.4% of participants in the HIT group and 76.4% in the MCT group adhered to at least 70% of the exercise sessions. The median weekly exercise duration was 96 min (IQR, 82–105) for HIT and 176 min (IQR, 137–188) for MCT. However, adherence decreased during the home phase (4–12 months) to 77 min per week (IQR, 46–92) for HIT and 144 min per week (IQR, 108–171) for MCT [49]. The HEART camp trial highlighted better long-term adherence in the home-based care group (HCG) compared to the usual care group (UCG). At 12 months, 42% of HCG participants adhered to the exercise regimen, which increased to 56% at 18 months. Conversely, adherence in the UCG dropped from 14% at 12 months to 0% at 18 months [51]. Another study found that 48 out of 58 HFpEF patients in the exercise training group (ETG) completed the final tests, achieving an adherence rate of 88% for the prescribed physical activities [50]. Fiatarone et al. had a mean adherence rate of 97 percent for RET in frail patients [76].

## 15. HFrEF Adherence Rates

A multicentre trial for HFrEF patients reported perfect adherence rates in both the HIT and MCT groups, with participants completing 100% of their sessions (35 out of 35 for HIT and 4 out of 4 for MCT) [58]. Additionally, a 16 week supervised ET programme showed a high adherence rate of 93.75%, with patients completing 45 out of 48 sessions [76].

A sub-study investigating predictors of adherence to ET programmes in HFrEF patients identified that a new diagnosis of heart failure significantly predicted adherence (*p* = 0.03) [77]. Meeting the exercise guideline (150 min per week of moderate exercise) at follow-up was more likely in those with a new diagnosis of heart failure (*p* = 0.0013) and those who were physically active at baseline (*p* = 0.007). The study concluded that individualised approaches are necessary to enhance adherence, particularly for decompensated heart failure patients and those who are initially inactive [76,77].

Further research is essential to develop effective supervision methods and improve long-term adherence rates of ET in both HFpEF and HFrEF patients.

## 16. Prescription Rates

There are generally poor prescription rates for ET in HF patients. Frail HF patients are unable to take part in vigorous exercise training, which may illicit a contributing factor to poor prescription and adherence rates. Patient motivation, socio-cultural factors, and burden of travel to cardiac rehabilitation centres are additional key factors affecting prescription rates. In a study of 513 patients with HFpEF and HFrEF, 100 percent were educated on the benefits of exercise; however, only 21 (4%) were enrolled in a supervised exercise programme [77]. A survey of 170 European cardiac centres found that 67 (39.4%) did not provide an ET programme [78]. This was mainly ascribed to a lack of resources and staff. In the US, out of 397,000 HFrEF Medicare patients eligible for an ET regimen, only 2.6% completed more than one training session in a 12 month timeframe [79]. Furthermore, of those prescribed cardiac rehabilitation, only 20% completed all of the sessions. Consequently, an accelerated shift into home-based cardiac rehabilitation programmes, including telerehabilitation, is an alternative intervention to mitigate these low rates and appears to demonstrate comparable effects on functional capacity and quality of life [80,81]. Nonetheless, further trials with robust outcome measures are required to support the widespread deployment of cardiac telerehabilitation.

## 17. Conclusions and Recommendations

Combining GDMT with exercise-based therapy has additional benefits for HF patients, improving functional capacity, exercise tolerance, and HRQOL outcomes. Prescription rates are low across different healthcare systems, and there are currently no well-established guidelines on establishing personalised regimens. The clinical characteristics (comorbidities, frailty status, exercise capacity, and personal needs) of patients are necessary when selecting patients for ET. However, the limitations of the current literature preclude broad recommendations at this period. Involvement of primary care in the long-term management of ET should be explored. This includes arranging follow-ups for the patients’ clinical status with questionnaires assessing QoL and adherence rates. Initial assessments should include selections of age-appropriate programmes, with ventilatory assistance offered to the advanced HF population. ET has been shown to yield some benefits in smaller trials; however, large robust trials are needed to allow us to draw definitive conclusions about the incorporation of ET in the treatment of HF patients. Until these have been fulfilled, a cautious interpretation of the presented benefits should be taken.

## 18. Limitations

Heterogeneity in study population, study design, and exercise-based cardiac rehabilitation–intervention was evident making this meta-analysis challenging. In addition, the trials reported have not adequately assessed the long-term effects of exercise training in the HF population. Insufficient data are reported on the hospitalisation and mortality rates following ET. Some studies reviewed did not include whether patients were established on GDMT. A limited number of studies reported QoL outcomes after ET. Further work is vital to address these limitations to aid efficacious exercise prescriptions for HF patients. Furthermore, many of the trials used small sample sizes, which proves to be a major limitation due to reduced generalisability, increased risk of bias, and reduced statistical power. This, along with short follow-up periods, hinder the extrapolation of results to broader patient populations. More robust large-scale trials are needed to draw definitive conclusions about the efficacy of exercise training in heart failure.

## Figures and Tables

**Table 1:** Efficacy of exercise training in HFpEF.

	Scaling Peak Oxygen Consumption for Body Size and Composition in People with a Fontan Circulation [13].	One-Year Committed Exercise Training Reverses Abnormal Left Ventricular Myocardial Stiffness in Patients with Stage B Heart Failure with Preserved Ejection Fraction [45].	High-Intensity Interval Training Is Effective and Superior to Moderate Continuous Training in Patients with Heart Failure with Preserved Ejection Fraction: A Randomised Clinical Trial [46].	Effect of High-Intensity Interval Training, Moderate Continuous Training, or Guideline-Based Physical Activity Advice on Peak Oxygen Consumption in Patients with Heart Failure with Preserved Ejection Fraction: A Randomised Clinical Trial [49].
Study type	Secondary data analysis.	RCT	RCT	RCT
Method	Ration + allometric scaling of VO_2peak_ to BM, fat-free mass, body surface area, and BM. (*n* = 89).	(*n* = 30) High-intensity exercise training or (*n* = 16) attention control.	HIIT (*n* = 10) vs. MCT (*n* = 9).	(*n* = 106) Exercise training ET vs. (*n* = 52) guideline control CON.
Results	Significant correlation between ratio scaled VO_2peak_ and BM (r = −0.25, *p* = 0.02), stature (r = 0.46, *p* < 0.001), and body surface area (r = 0.23, *p* = 0.03), and not with fat-free mass (r ≤ 0.11; *R*^2^ = 1%). No significant correlation between allometrically expressed VO_2peak_ and any scaling denominator were not (*r* ≤ 0.11; *R*^2^ = 1%).	Significant increase in VO_2_ max with HIIT and LVEDV (*p* < 0.0001). No sign of significant change in VO_2_ max or LVEDV (*p* = 0.175) in controls. Unchanged resting BP in both groups. LV myocardial stiffness reduced with HIIT. No statistically significant change in controls.	Significant increase in VO_2peak_ in both groups (HIIT 22.7%, MCT 11.3% at *p* < 0.001). HIIT group increased peak oxygen pulse and estimates of stroke volume further. First ventilatory anaerobic threshold increased in both groups (12.1 ± 0.6 increased to 13.4 ± 0.7 in MCT 11.5 ± 0.8 increased to 12.6 ± 0.8 mL·kg^−1^·min^−1^ in HIIT). No difference in peak RER between groups.	Relative peak VO_2_ increased by 8.0 ± 15.7% in the ET group. Relative peak reduced in the CON group by −2.0 ± 18.3%. Differences in the change between groups were primarily mediated by changes in peak O_2_-pulse (~72%). Significantly different mean changes between groups for weight, relative peak VO_2_, absolute peak VO_2_, and peak O_2_-pulse. No significant differences in change between peak HR, haemoglobin, or peak respiratory exchange ratio (RER).
	Exercise training effects on the relationship of physical function and health-related quality of life among older heart failure patients with preserved ejection fraction [50].	The HEART camp exercise intervention improves exercise adherence, physical function, and patient-reported outcomes in adults with preserved ejection fraction heart failure [51].	A randomised controlled trial of a facilitated home-based rehabilitation intervention in patients with heart failure with preserved ejection fraction and their caregivers: the REACH-HFpEF Pilot Study [54].	The impact of a nurse-led care programme on events and physical and psychosocial parameters in patients with heart failure with preserved ejection fraction: a randomised clinical trial in primary care in Russia [55].
Study Type	RCT	RCT	RCT	RCT
Method	Endurance training ET (*n* = 58) vs. attention control CON (*n* = 58).	HEART camp (*n* = 25) vs. enhanced usual care (*n* = 34).	REACH-HF intervention group + usual care (patients *n* = 25, caregivers *n* = 11) or usual care alone (patients *n* = 25, caregivers *n* = 10)	Nurse-led patient education and care plan intervention (*n* = 44) vs. usual care (*n* = 41).
Results	ET group had a significant improvement in VO_2peak,_ VAT, 6MWT, and peak exercise test workload compared to CON at *p* < 0.001. Significant improvement in SF-36 components: Physical composite (*p* = 0.03); Role physical (*p* = 0.006); Vitality (*p* = 0.001). No difference in mental health sub-scores. No significant differences in MLHF scores. No correlation between physical function and HRQOL score.	HEART camp group had a significantly improved 6MWT compared to controls. Distance in the HEART Camp group increased by 63.25 m. Distance in the usual care group increased by 13.16 m. Significant difference in the KCCQ Overall Summary Score: (F(3, 96) = 3.42, *p* = 0.02; η^2^ = 0.09, medium effect). The Clinical Summary Score: (F(3, 95) = 6.17, *p* = 0.001; η^2^ = 0.16, large effect). Total Symptom Score: (F(2.7, 85.3) = 5.31, *p* = 0.003; η^2^ = 0.14, large effect) between groups.	At 6 months, the data potentially show that intervention is favourable to patient outcomes, including Minnesota Living with Heart Failure Questionnaire total score (between-group mean difference −11.5), HeartQoL Global Score (0.5), EQ-5D-3L Utility Index (0.11), HADS Depression Score (−1.5), and SCHFI Maintenance Score (9.5). In total, 11 patient hospital admission; 4 of these are control admissions being HF-related over 6 months.	Intervention group significantly improved BMI, waist circumference, 6MWT, level of anxiety, total cholesterol, LDT, LVEDV index, and QoL. There were 11 deaths (25%) or hospitalisations in the intervention group and 12 (29%) in the control group over 18 months. No significant difference in risk of cardiovascular events at 6 months (HR = 0.47) or 18 months (HR = 0.85).

**Table 2:** Efficacy of exercise training in HFrEF.

	Response to Endurance Exercise Training in Older Adults with Heart Failure with Preserved or Reduced Ejection Fraction [52].	Efficacy and Safety of Exercise Training in Patients with Chronic Heart Failure HF-ACTION in a Randomised Controlled Trial [43].	Short-Term Effects of a 3-Week Interval Training Programme on Heart Rate Variability in Chronic Heart Failure. Randomised Controlled Trial Short-Term Effects of a 3 Week Interval Training Programme on Heart Rate Variability in Chronic Heart Failure. A Randomised Controlled Trial [56].	High-Intensity Interval Training in Patients with Heart Failure with Reduced Ejection Fraction [57].	Combined Aerobic/Resistance/Inspiratory Muscle Training as the ‘Optimum’ Exercise Programme for Patients with Chronic Heart Failure: Aristos-HF Randomised Clinical Trial [58].
Study type	Secondary analysis of an RCT	RCT	RCT	RCT	RCT
Method arms	In total, 24 HFrEF and 24 HFpEF individuals who underwent supervised exercise training.	Usual care plus aerobic exercise training (*n* = 1159) vs. usual care alone (*n* = 1172).	MICT (*n* = 15) vs. HIIT (*n* = 16).	HIIT, (*n* = 77), MCT (*n* = 65), vs. recommendation of regular exercise (RRE) (*n* = 73).	ARIS (*n* = 19) vs. AT/IMT (*n* = 20) vs. AT/RT (*n* = 17) vs. AT (*n* = 18).
Results	Endurance training had a 9.2% increase in VO_2peak_ for both groups combined with significant individual-level variability in the change in response to training. Improvement in VO_2peak_ substantially higher in HFpEF vs. HFrEF patients (18.7 ± 17.6 vs. −0.3 ± 15.4%; *p* < 0.001). Absolute VO_2peak_ saw a similar trend. Other measures of exercise capacity were also greater in HFpEF vs. HFrEF patients, trending towards statistical significance. There was little to no significant difference changes in 6MWT between groups found.	In total, 759 (65%) patients in the exercise group died or were hospitalised, compared to 796 (68%) in the usual care group (hazard ratio [HR], 0.93; 95% CI 0.84–1.02; *p* = 0.13). Nonsignificant reductions in the exercise group for mortality were seen: - 189 [16%] in the exercise group; - 198 [17%] in the usual care group; - HR 0.96. Cardiovascular mortality or cardiovascular hospitalisation: - 632 [55%] in the exercise group; - 677 [58%] in the usual care group; - HR 0.92. Cardiovascular mortality or heart failure hospitalisation: - 344 (30%) in the exercise group; - 393 (34%) in the usual care group; - HR, 0.87. Other adverse events were similar between the groups.	High-frequency power in normalised units (HFnu%) increased with HIIT (from 21.2% to 26.4%, *p* < 0.001), but with MICT, it remained unchanged (from 23.1% to 21.9%, *p =* 0.444). However, there was a significant intergroup difference, *p =* 0.003. Resting heart rate decreased for both groups (from 68.2 to 64.6 bpm and 66.0 to 63.5 bpm for MICT and HIIT, respectively, with no intergroup difference, *p =* 0.578) No difference in premature ventricular contractions Improvement in peak oxygen uptake was greater with HIIT than MICT (+21% vs. +5%, *p =* 0.009) LVEF improved with only HIIT (from 36.2% to 39.5%, *p =* 0.034).	Change in LVED diameter from baseline to 12 weeks was not different (*p* = 0.45); LVED diameter changes compared to RRE were −2.8 mm (−5.2 to −0.4 mm; *p* = 0.02) in HIIT and −1.2 mm (−3.6 to 1.2 mm; *p* = 0.34) in MCT. No difference between the two groups (HIIT and MCT) in peak oxygen uptake (*p* = 0.70), but it was evident that both were better than RRE. No changes maintained after 52 weeks. Serious adverse events were not different at any time (HIIT, 39%; MCT, 25%; RRE, 34%; *p* = 0.16).	Trends show increased peakVO_2_ (mL/kg/min) [mean contrasts (95% CI)] in the ARIS group [ARIS vs. AT/RT 1.71 (0.163–3.25), vs. AT/IMT 1.50 (0.0152–2.99), vs. AT 1.38 (−0.142 to 2.9)]. Increased LVES diameter (mm) [ARIS vs. AT/RT −2.11 (−3.65 to (−0.561)), vs. AT −2.47 (−4.01 to (−0.929))]; 6MWT (m) [ARIS vs. AT/IMT 45.6 (18.3–72.9), vs. AT 55.2 (27.6–82.7)].
	Combined endurance/resistance training reduces NT-probnp levels in patients with chronic heart failure [59].	Home-based cardiac rehabilitation improves quality of life, aerobic capacity, and readmission rates in patients with chronic heart failure [60].		Effect of stress related neural pathways on cardiovascular benefit of physical activity [64].	
Study Type	Non-RCT	RCT			
Methods	Resistance and endurance training (*n* = 27) vs. untrained (*n* = 22).	Control group (*n* = 18) vs. interventional group (*n* = 19). Within the interventional group included 3 months of individualised rehabilitation programmes, including home-based cardiac rehabilitation, diet education, and management of daily activity.		In total, 50,359 participants from the Mass General Brigham Biobank completed a physical activity (PA) survey were studied. A subset underwent ^18^FDG PET/CT imaging. Stress-related neural activity measured as resting amygdalar-to-cortical activity ratio (AmygA_C_). CVD events were determined from electronic health records.	
Results	Training reduced NT-proBNP levels (2124 ± 97 pg/mL before, 1635 ± 04 pg/mL after training, *p* = 0.015; *p* = 0.046, interaction) and improved NYHA functional class (from 2.8 ± 0.1 to 2.3 ± 0.1, *p* = 0.0002; *p* = 0.02, interaction). Both remained unchanged in control group (*p* = 0.9 for NT-proBNP). At 4 months there was significant increase in maximal VO_2peak_ +2.0 mL/kg^−1^/min.	The intervention group had statistically significant improvement in VO_2peak_ (18.2 ± 4.1 vs. 20.9 ± 6.6 mL/kg/min, *p* = 0.02), Maximal 6MWD (421 ± 90 vs. 462 ± 74 m, *p* = 0.03), Anaerobic threshold (12.4 ± 2.5 vs. 13.4 ± 2.6 mL/kg/min, *p* = 0.005), and QoL. A 14.2% increase in VO_2peak_, 37% increase in QoL score, and 41m improvement on the 6MWD test in the intervention group. The 90 day readmission rate for intervention patients reduced from 14% to 5%.		Greater PA associated with lower AmygA_C_ (standardised β: −0.245; 95% CI: −0.444 to −0.046; *p* = 0.016) in multi-variable models. Greater PA associated with lower CVD events (HR: 0.802; 95% CI: 0.719–0.896; *p* < 0.001) in multi-variable models. AmygA_C_ reductions partially mediated PA’s CVD benefit (OR: 0.96; 95% CI: 0.92–0.99; *p* < 0.05). Greater benefit of PA on incident CVD events for those with (vs. without) pre-existing depression (HR: 0.860 vs. HR: 0.929, *p* interaction = 0.011).	

## References

[1] Savarese G., Becher P.M., Lund L.H., Seferovic P., Rosano G.M.C., Coats A.J.S. (2023). Global burden of heart failure: A comprehensive and updated review of epidemiology. Cardiovasc. Res..

[2] Smeets M., Vaes B., Mamouris P., Van Den Akker M., Van Pottelbergh G., Goderis G., Janssens S., Aertgeerts B., Henrard S. (2019). Burden of heart failure in Flemish general practices: A registry-based study in the Intego database. BMJ Open.

[3] van Riet E.E., Hoes A.W., Wagenaar K.P., Limburg A., Landman M.A., Rutten F.H. (2016). Epidemiology of heart failure: The prevalence of heart failure and ventricular dysfunction in older adults over time. A systematic review. Eur. J. Heart Fail..

[4] Redfield M.M., Borlaug B.A. (2023). Heart failure with preserved ejection fraction. J. Am. Med. Assoc..

[5] Savarese G., Gatti P., Benson L., Adamo M., Chioncel O., Crespo-Leiro M.G., Anker S.D., Coats A.J., Filippatos G., Lainscak M. (2024). Left ventricular ejection fraction digit bias and reclassification of heart failure with mildly reduced vs reduced ejection fraction based on the 2021 definition and classification of heart failure. Am. Heart J..

[6] McDonagh T.A., Metra M., Adamo M., Gardner R.S., Baumbach A., Böhm M., Burri H., Butler J., Čelutkienė J., Chioncel O. (2023). 2023 Focused Update of the 2021 ESC Guidelines for the diagnosis and treatment of acute and chronic heart failure: Developed by the task force for the diagnosis and treatment of acute and chronic heart failure of the European Society of Cardiology (ESC) With the special contribution of the Heart Failure Association (HFA) of the ESC. Eur. Heart J..

[7] Chioncel O., Lainscak M., Seferovic P.M., Anker S.D., Crespo-Leiro M.G., Harjola V.P., Parissis J., Laroche C., Piepoli M.F., Fonseca C. (2017). Epidemiology and one-year outcomes in patients with chronic heart failure and preserved, mid-range and reduced ejection fraction: An analysis of the ESC Heart Failure Long-Term Registry. Eur. J. Heart Fail..

[8] Bauer J., Morley J.E., Schols A., Ferrucci L., Cruz-Jentoft A.J., Dent E., Baracos V.E., Crawford J.A., Doehner W., Heymsfield S.B. (2019). Sarcopenia: A Time for Action. An SCWD Position Paper. J. Cachexia Sarcopenia Muscle.

[9] Emami A., Saitoh M., Valentova M., Sandek A., Evertz R., Ebner N., Loncar G., Springer J., Doehner W., Lainscak M. (2018). Comparison of sarcopenia and cachexia in men with chronic heart failure: Results from the Studies Investigating Co-morbidities Aggravating Heart Failure (SICA-HF). Eur. J. Heart Fail..

[10] Fülster S., Tacke M., Sandek A., Ebner N., Tschöpe C., Doehner W., Anker S.D., von Haehling S. (2013). Muscle wasting in patients with chronic heart failure: Results from the studies investigating co-morbidities aggravating heart failure (SICA-HF). Eur. Heart J..

[11] Adams V., Linke A., Winzer E. (2017). Skeletal muscle alterations in HFrEF vs. HFpEF. Curr. Heart Fail. Rep..

[12] Wing S.S., Lecker S.H., Jagoe R.T. (2011). Proteolysis in illness-associated skeletal muscle atrophy: From pathways to networks. Crit. Rev. Clin. Lab. Sci..

[13] Wadey C.A., Barker A.R., Stuart G., Tran D.L., Laohachai K., Ayer J., Cordina R., Williams C.A. (2022). Scaling Peak Oxygen Consumption for Body Size and Composition in People With a Fontan Circulation. J. Am. Heart Assoc..

[14] Juarez M., Castillo-Rodriguez C., Soliman D., Del Rio-Pertuz G., Nugent K. (2024). Cardiopulmonary Exercise Testing in Heart Failure. J. Cardiovasc. Dev. Dis..

[15] Rostagno C., Gensini G.F. (2008). Six minute walk test: A simple and useful test to evaluate functional capacity in patients with heart failure. Intern. Emerg. Med..

[16] Olsson L.G., Swedberg K., Clark A.L., Witte K.K., Cleland J.G. (2005). Six minute corridor walk test as an outcome measure for the assessment of treatment in randomized, blinded intervention trials of chronic heart failure: A systematic review. Eur. Heart J..

[17] Opasich C., Pinna G.D., Mazza A., Febo O., Riccardi R., Riccardi P.G., Capomolla S., Forni G., Cobelli F., Tavazzi L. (2001). Six-minute walking performance in patients with moderate-to-severe heart failure; is it a useful indicator in clinical practice?. Eur. Heart J..

[18] Bittner V., Weiner D.H., Yusuf S., Rogers W.J., McIntyre K.M., Bangdiwala S.I., Kronenberg M.W., Kostis J.B., Kohn R.M., Guillotte M. (1993). Prediction of mortality and morbidity with a 6-minute walk test in Patients With Left Ventricular Dysfunction. J. Am. Med. Assoc..

[19] Denfeld Q.E., Winters-Stone K., Mudd J.O., Gelow J.M., Kurdi S., Lee C.S. (2017). The prevalence of frailty in heart failure: A systematic review and meta-analysis. Int. J. Cardiol..

[20] Bielecka-Dabrowa A., Ebner N., Dos Santos M.R., Ishida J., Hasenfuss G., von Haehling S. (2020). Cachexia, muscle wasting, and frailty in cardiovascular disease. Eur. J. Heart Fail..

[21] Vidán M.T., Blaya-Novakova V., Sánchez E., Ortiz J., Serra-Rexach J.A., Bueno H. (2016). Prevalence and prognostic impact of frailty and its components in non-dependent elderly patients with heart failure. Eur. J. Heart Fail..

[22] Dewan P., Jackson A., Jhund P.S., Shen L., Ferreira J.P., Petrie M.C., Abraham W.T., Desai A.S., Dickstein K., Køber L. (2020). The prevalence and importance of frailty in heart failure with reduced ejection fraction—An analysis of PARADIGM-HF and ATMOSPHERE. Eur. J. Heart Fail..

[23] Sanders N.A., Supiano M.A., Lewis E.F., Liu J., Claggett B., Pfeffer M.A., Desai A.S., Sweitzer N.K., Solomon S.D., Fang J.C. (2018). The frailty syndrome and outcomes in the TOPCAT trial. Eur. J. Heart Fail..

[24] Fernandes B.P., Conceição L.S., Martins-Filho P.R., de Santana Motta D.R., Carvalho V.O. (2018). Effect of mineralocorticoid receptor antagonists in individuals with heart failure with preserved Ejection Fraction: A systematic review. J. Card. Fail..

[25] Pagel P.S., Tawil J.N., Boettcher B.T., Izquierdo D.A., Lazicki T.J., Crystal G.J., Freed J.K. (2021). Heart Failure With Preserved Ejection Fraction: A Comprehensive Review and Update of Diagnosis, Pathophysiology, Treatment, and Perioperative Implications. J. Cardiothorac. Vasc. Anesth..

[26] Gayat E., Arrigo M., Littnerova S., Sato N., Parenica J., Ishihara S., Spinar J., Müller C., Harjola V.P., Lassus J. (2018). Heart failure oral therapies at discharge are associated with better outcome in acute heart failure: A propensity-score matched study. Eur. J. Heart Fail..

[27] Crespo-Leiro M.G., Anker S.D., Maggioni A.P., Coats A.J., Filippatos G., Ruschitzka F., Ferrari R., Piepoli M.F., Delgado Jimenez J.F., Metra M. (2016). European Society of Cardiology Heart Failure Long-Term Registry (ESC-HF-LT): 1-year follow-up outcomes and differences across regions. Eur. J. Heart Fail..

[28] McMurray J.J., Packer M., Desai A.S., Gong J., Lefkowitz M.P., Rizkala A.R., Rouleau J.L., Shi V.C., Solomon S.D., Swedberg K. (2014). Angiotensin-neprilysin inhibition versus enalapril in heart failure. N. Engl. J. Med..

[29] Dunlay S., Roger V., Redfield M. (2017). Epidemiology of heart failure with preserved ejection fraction. Nat. Rev. Cardiol..

[30] McMurray JJ V., Solomon S.D., Inzucchi S.E., Køber L., Kosiborod M.N., Martinez F.A., Ponikowski P., Sabatine M.S., Anand I.S., Bělohlávek J. (2019). Dapagliflozin in Patients with Heart Failure and Reduced Ejection Fraction. N. Engl. J. Med..

[31] Lv J., Li Y., Shi S., Xu X., Wu H., Zhang B., Song Q. (2022). Skeletal muscle mitochondrial remodeling in heart failure: An update on mechanisms and therapeutic opportunities. Biomed. Pharmacother..

[32] Drexler H. (1992). Effect of angiotensin-converting enzyme inhibitors on the peripheral circulation in heart failure. Am. J. Cardiol..

[33] Malfatto G., Ravaro S., Caravita S., Baratto C., Sorropago A., Giglio A., Villani A. (2019). Improvement of functional capacity in sacubitril-valsartan treated patients assessed by cardiopulmonary exercise test. Acta Cardiol..

[34] Campanile A., Visco V., De Carlo S., Ferruzzi G.J., Mancusi C., Izzo C., Mongiello F., Di Pietro P., Virtuoso N., Ravera A. (2023). Sacubitril/Valsartan vs. Standard Medical Therapy on Exercise Capacity in HFrEF Patients. Life.

[35] Romano G., Vitale G., Ajello L., Agnese V., Bellavia D., Caccamo G., Corrado E., Di Gesaro G., Falletta C., La Franca E. (2019). The Effects of Sacubitril/Valsartan on Clinical, Biochemical and Echocardiographic Parameters in Patients with Heart Failure with Reduced Ejection Fraction: The “Hemodynamic Recovery”. J. Clin. Med..

[36] Berbenetz N.M., Mrkobrada M. (2016). Mineralocorticoid receptor antagonists for heart failure: Systematic review and meta-analysis. BMC Cardiovasc. Disord..

[37] Heidenreich P.A., Bozkurt B., Aguilar D., Allen L.A., Byun J.J., Colvin M.M., Deswal A., Drazner M.H., Dunlay S.M., Evers L.R. (2022). 2022 AHA/ACC/HFSA guideline for the management of heart failure: A report of the American College of Cardiology/American Heart Association Joint Committee on Clinical Practice Guidelines. Circulation.

[38] Anker S.D., Butler J., Filippatos G., Ferreira J.P., Bocchi E., Böhm M., Brunner-La Rocca H.P., Choi D.J., Chopra V., Chuquiure-Valenzuela E. (2021). EMPEROR-Preserved Trial Investigators Empagliflozin in Heart Failure with a Preserved Ejection Fraction. N. Engl. J. Med..

[39] Dankowski R., Kotwica T., Szyszka A., Przewłocka-Kosmala M., Sacharczuk W., Karolko B., Kobusiak-Prokopowicz M., Mysiak A., Kosmala W. (2018). Determinants of the beneficial effect of mineralocorticoid receptor antagonism on exercise capacity in heart failure with reduced ejection fraction. Kardiol. Pol..

[40] Palau P., Seller J., Domínguez E., Sastre C., Ramón J.M., de La Espriella R., Santas E., Miñana G., Bodí V., Sanchis J. (2021). Effect of β-Blocker Withdrawal on Functional Capacity in Heart Failure and Preserved Ejection Fraction. J. Am. Coll. Cardiol..

[41] Awad A.K., Abdelgalil M.S., Gonnah A.R., Mouffokes A., Ahmad U., Awad A.K., Elbadawy M.A., Roberts D.H. (2024). Intravenous Iron for Acute and Chronic Heart Failure with Reduced Ejection Fraction (HFrEF) Patients with Iron Deficiency: An Updated Systematic Review and Meta-Analysis. Clin. Med..

[42] Pina I.L., Apstein C.S., Balady G.J., Belardinelli R., Chaitman B.R., Duscha B.D., Fletcher B.J., Fleg J.L., Myers J.N., Sullivan M.J. (2003). Exercise and heart failure: A statement from the American Heart Association Committee on Exercise, Rehabilitation, and Prevention. Circulation.

[43] O’Connor C.M., Whellan D.J., Lee K.L., Keteyian S.J., Cooper L.S., Ellis S.J., Leifer E.S., Kraus W.E., Kitzman D.W., Blumenthal J.A. (2009). Efficacy and safety of exercise training in patients with chronic heart failure: HF-ACTION randomized controlled trial. J. Am. Med. Assoc..

[44] Forman D.E., Sanderson B.K., Josephson R.A., Raikhelkar J., Bittner V., American College (2015). Heart failure as a newly approved diagnosis for cardiac rehabilitation: Challenges and opportunities. J. Am. Coll. Cardiol..

[45] Hieda M., Sarma S., Hearon C.M., MacNamara J.P., Dias K.A., Samels M., Palmer D., Livingston S., Morris M., Levine B. (2021). One-Year Committed Exercise Training Reverses Abnormal Left Ventricular Myocardial Stiffness in Patients With Stage B Heart Failure With Preserved Ejection Fraction. Circulation.

[46] da Silveira A.D., de Lima J.B., Piardi D.d.S., Macedo D.d.S., Zanini M., Nery R., Laukkanen J.A., Stein R. (2020). High-intensity interval training is effective and superior to moderate continuous training in patients with heart failure with preserved ejection fraction: A randomized clinical trial. Eur. J. Prev. Cardiol..

[47] NICE. 2018. Chronic Heart Failure in Adults: Diagnosis and Management. NICE Guideline [NG106]. https://www.nice.org.uk/guidance/ng106.

[48] Shabeer H., Samore N., Ahsan S., Gondal MU R., Shah B.U.D., Ashraf A., Faraz M., Malik J. (2024). Safety and Efficacy of Ferric Carboxymaltose in Heart Failure With Preserved Ejection Fraction and Iron Deficiency. Curr. Probl. Cardiol..

[49] Mueller S., Winzer E.B., Duvinage A., Gevaert A.B., Edelmann F., Haller B., Pieske-Kraigher E., Beckers P., Bobenko A., Hommel J. (2021). Effect of High-Intensity Interval Training, Moderate Continuous Training, or Guideline-Based Physical Activity Advice on Peak Oxygen Consumption in Patients With Heart Failure With Preserved Ejection Fraction: A Randomized Clinical Trial. J. Am. Med. Assoc..

[50] Brubaker P.H., Avis T., Rejeski W.J., Mihalko S.E., Tucker W.J., Kitzman D.W. (2020). Exercise Training Effects on the Relationship of Physical Function and Health-Related Quality of Life Among Older Heart Failure Patients With Preserved Ejection Fraction. J. Cardiopulm. Rehabil. Prev..

[51] Alonso W.W., Kupzyk K.A., Norman J.F., Lundgren S.W., Fisher A., Lindsey M.L., Keteyian S.J., Pozehl B.J. (2022). The HEART Camp Exercise Intervention Improves Exercise Adherence, Physical Function, and Patient-Reported Outcomes in Adults With Preserved Ejection Fraction Heart Failure. J. Card. Fail..

[52] Pandey A., Kitzman D.W., Brubaker P., Haykowsky M.J., Morgan T., Becton J.T., Berry J.D. (2017). Response to Endurance Exercise Training in Older Adults with Heart Failure with Preserved or Reduced Ejection Fraction. J. Am. Geriatr. Soc..

[53] Jankowska E.A., Wegrzynowska K., Superlak M., Nowakowska K., Lazorczyk M., Biel B., Kustrzycka-Kratochwil D., Piotrowska K., Banasiak W., Wozniewski M. (2008). The 12-week progressive quadriceps resistance training improves muscle strength, exercise capacity and quality of life in patients with stable chronic heart failure. Int. J. Cardiol..

[54] Lang C.C., Smith K., Wingham J., Eyre V., Greaves C.J., Warren F.C., Green C., Jolly K., Davis R.C., Doherty P.J. (2018). A randomised controlled trial of a facilitated home-based rehabilitation intervention in patients with heart failure with preserved ejection fraction and their caregivers: The REACH-HFpEF Pilot Study. BMJ Open.

[55] Andryukhin A., Frolova E., Vaes B., Degryse J. (2010). The impact of a nurse-led care programme on events and physical and psychosocial parameters in patients with heart failure with preserved ejection fraction: A randomized clinical trial in primary care in Russia. Eur. J. Gen. Pract..

[56] Besnier F., Labrunée M., Richard L., Faggianelli F., Kerros H., Soukarié L., Bousquet M., Garcia J.-L., Pathak A., Gales C. (2019). Short-term effects of a 3-week interval training program on Heart Rate Variability in chronic heart failure. A randomised controlled trial. Ann. Phys. Rehabil. Med..

[57] Ellingsen Ø., Halle M., Conraads V., Støylen A., Dalen H., Delagardelle C., Larsen A.-I., Hole T., Mezzani A., Van Craenenbroeck E.M. (2017). High-Intensity Interval Training in Patients With Heart Failure With Reduced Ejection Fraction. Circulation.

[58] Laoutaris I.D., Piotrowicz E., Kallistratos M.S., Dritsas A., Dimaki N., Miliopoulos D., Andriopoulou M., Manolis A.J., Volterrani M., Piepoli M.F. (2020). Combined aerobic/resistance/inspiratory muscle training as the ‘optimum’ exercise programme for patients with chronic heart failure: Aristos-HF randomized clinical trial. Eur. J. Prev. Cardiol..

[59] Conraads V., Beckers P., Vaes J., Martin M., Vanhoof V., Demaeyer C., Possemiers N., Wuyts F.L., Vrints C.J. (2004). Combined endurance/resistance training reduces NT-probnp levels in patients with chronic heart failure. Eur. Heart J..

[60] Chen Y.W., Wang C.Y., Lai Y.H., Liao Y.C., Wen Y.K., Chang S.T., Huang J.L., Wu T.J. (2018). Home-based cardiac rehabilitation improves quality of life, aerobic capacity, and readmission rates in patients with chronic heart failure. Medicine.

[61] Tcheugui J.B., Zhang S., McEvoy J.W., Ndumele C.E., Hoogeveen R.C., Coresh J., Selvin E. (2022). Elevated NT-ProBNP as a Cardiovascular Disease Risk Equivalent: Evidence from the Atherosclerosis Risk in Communities (ARIC) Study. Am. J. Med..

[62] Rørth R., Jhund P.S., Yilmaz M.B., Kristensen S.L., Welsh P., Desai A.S., Køber L., Prescott M.F., Rouleau J.L., Solomon S.D. (2020). Comparison of BNP and NT-proBNP in Patients With Heart Failure and Reduced Ejection Fraction. Circ. Heart Fail..

[63] Hall C. (2005). NT-ProBNP: The mechanism behind the marker. J. Card. Fail..

[64] Zureigat H., Osborne M., Abohashem S., Mezue K., Gharios C., Grewal S., Cardeiro A., Naddaf N., Civieri G., Abbasi T. (2024). Effect of Stress-Related Neural Pathways on the Cardiovascular Benefit of Physical Activity. J. Am. Coll. Cardiol..

[65] Moser D.K., Dickson V., Jaarsma T., Lee C., Stromberg A., Riegel B. (2012). Role of self-care in the patient with heart failure. Curr. Cardiol. Rep..

[66] Fiatarone M.A., O’Neill E.F., Ryan N.D., Clements K.M., Solares G.R., Nelson M.E., Roberts S.B., Kehayias J.J., Lipsitz L.A., Evans W.J. (1994). Exercise training and nutritional supplementation for physical frailty in very elderly people. N. Engl. J. Med..

[67] Kuhn B.T., Bradley L.A., Dempsey T.M., Puro A.C., Adams J.Y. (2016). Management of Mechanical Ventilation in Decompensated Heart Failure. J. Cardiovasc. Dev. Dis..

[68] O’Donnell D.E., D’Arsigny C., Raj S., Abdollah H., Webb K.A. (1999). Ventilatory assistance improves exercise endurance in stable congestive heart failure. Am. J. Respir. Crit. Care Med..

[69] Caraballo C., Desai N.R., Mulder H., Alhanti B., Wilson F.P., Fiuzat M., Felker G.M., Piña I.L., O’Connor C.M., Lindenfeld J. (2019). Clinical Implications of the New York Heart Association Classification. J. Am. Heart Assoc..

[70] University of Minnesota (2024). Available Technologies. Minnesota LIVING WITH HEART FAILURE Questionnaire (MLHFQ).

[71] Spertus J.A., Jones P.G., Sandhu A.T., Arnold S.V. (2020). Interpreting the Kansas City Cardiomyopathy Questionnaire in clinical trials and clinical care. J. Am. Coll. Cardiol..

[72] Ware J.E., Sherbourne C.D. (1992). The MOS 36-Item Short-Form Health Survey (SF-36): I. Conceptual Framework and Item Selection. Med. Care.

[73] Norman J.F., Kupzyk K.A., Artinian N.T., Keteyian S.J., Alonso W.S., Bills S.E., Pozehl B.J. (2020). The influence of the HEART Camp intervention on physical function, health-related quality of life, depression, anxiety and fatigue in patients with heart failure. Eur. J. Cardiovasc. Nurs..

[74] Chrysohoou C., Tsitsinakis G., Vogiatzis I., Cherouveim E., Antoniou C., Tsiantilas A., Tsiachris D., Dimopoulos D., Panagiotakos D.B., Pitsavos C. (2013). High intensity, interval exercise improves quality of life of patients with chronic heart failure: A randomized controlled trial. QJM Int. J. Med..

[75] Villareal D.T., Aguirre L., Gurney A.B., Waters D.L., Sinacore D.R., Colombo E., Armamento-Villareal R., Qualls C. (2017). Aerobic or Resistance Exercise, or Both, in Dieting Obese Older Adults. N. Engl. J. Med..

[76] Brubaker P.H., Moore J.B., Stewart K.P., Wesley D.J., Kitzman D.W. (2009). Endurance exercise training in older patients with heart failure: Results from a randomized, controlled, single-blind trial. J. Am. Geriatr. Soc..

[77] Guha K., Allen C.J., Chawla S., Pryse-Hawkins H., Fallon L., Chambers V., Vazir A., Lyon A.R., Cowie M.R., Sharma R. (2016). Audit of a tertiary heart failure outpatient service to assess compliance with NICE guidelines. Clin. Med..

[78] Piepoli M.F., Binno S., Corrà U., Seferovic P., Conraads V., Jaarsma T., Schmid J., Filippatos G., Ponikowski P.P., Committee on Exercise Physiology & Training of the Heart Failure Association of the ESC (2015). ExtraHF survey: The first European survey on implementation of exercise training in heart failure patients. Eur. J. Heart Fail..

[79] Keteyian S.J., Michaels A. (2022). Heart failure in cardiac rehabilitation. J. Cardiopulm. Rehabil. Prev..

[80] Chien C.L., Lee C.M., Wu Y.W., Wu Y.T. (2011). Home-based exercise improves the quality of life and physical function but not the psychological status of people with chronic heart failure: A randomised trial. J. Physiother..

[81] Hwang R., Bruning J., Morris N.R., Mandrusiak A., Russell T. (2017). Home-based telerehabilitation is not inferior to a centre-based program in patients with chronic heart failure: A randomised trial. J. Physiother..

